# Supplementary material for: Single-molecule visualization of stalled replication-fork rescue by the Escherichia coli Rep helicase
Source: Nucleic Acids Res. 2023 Mar 20;51(7):3307–26. doi: 10.1093/nar/gkad186 (PMC10123100; doi:10.1093/nar/gkad186)
Supplement: gkad186_Supplemental_File [file gkad186_supplemental_file.pdf]

Single-molecule visualization of stalled replication-fork rescue by the *Escherichia coli* Rep helicase

Kelsey S. Whinn[1,2], Zhi-Qiang Xu[1,2], Slobodan Jergic[1,2], Nischal Sharma[1,2], Lisanne M. Spenkelink[1,2], Nicholas E. Dixon[1,2], Antoine M. van Oijen[1,2,*] and Harshad Ghodke[1,2]

[1] Molecular Horizons and School of Chemistry and Molecular Bioscience, University of Wollongong, Wollongong, New South Wales 2522, Australia

[2] Illawarra Health & Medical Research Institute, Wollongong, New South Wales 2522, Australia

* To whom correspondence should be addressed. Tel: +61 2 4221 4780; Email: vanoijen@uow.edu.au

Supplementary Table S1: Nucleic acid substrates used in this study.

Experiment	Substrate	Sequence
Trap dsDNA/ 83mer	83_S	5′-CAC ATG CTA TGA GCT GTT GCA ATC TCT CGT ACA ATT AAT AGA CTG GAT GGT GGA TGA CAA AGC TCT ACA CTA GAT ACT CAC AC-3′
	83_AS	3-bio-GTG TAC GAT ACT CGA CAA CGT TAG AGA GCA TGT TAA TTA TCT GAC CTA CCA CCT ACT GTT TCG AGA TGT GAT CTA TGA GTG TG-5′
gRNA	cgRNA1	5′ - ACA AUU AAU AGA CUG GAU GG
	cgRNA3	5′ - CAU UCC UGC AGC GAG UCC AU
	cgRNA4	5′ - AAA CUC ACG UUA AGG GAU UU
Mismatch gRNA	gRNA80	5′ - cac cUU AAU AGA CUG GAU GG
	gRNA60	5′ - cac cgg ccU AGA CUG GAU GG
	gRNA40	5′ - cac cgg ccg cuc CUG GAU GG
	gRNA20	5′ - cac cgg ccg cuc agu uAU GG

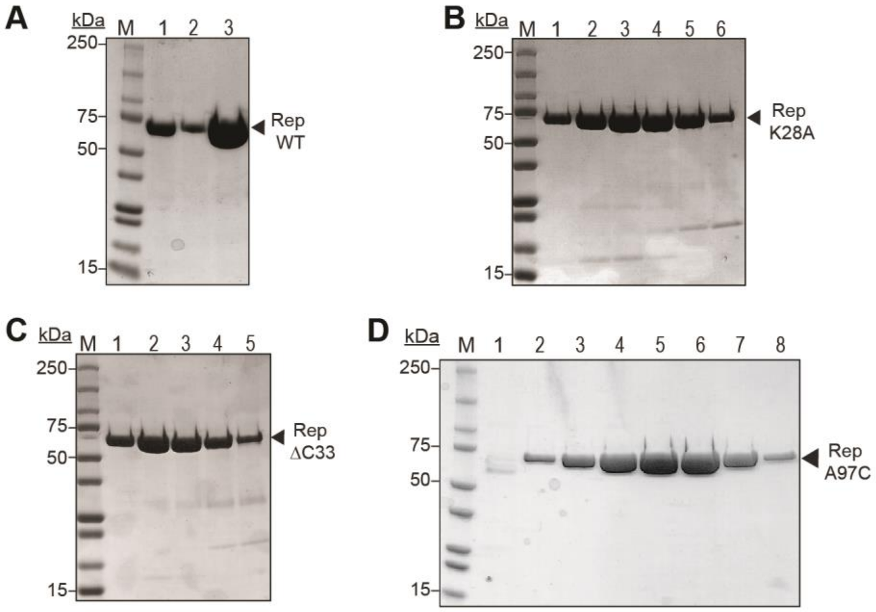

Supplementary Figure S1: Purification of Rep proteins using 5 mL HiTrap Heparin columns. Samples were analyzed on 4–20% SDS-PAGE gels. **(A)** Purification of His_6 Rep WT. Sample after purification on His-Trap column (lane 1), fractions of purified protein from Heparin column (lanes 2–3) used in this study. **(B)** Purification of His_6 Rep K28A. Samples from successive fractions (indicated by lane numbers 1–6). **(C)** Purification of His_6 Rep ΔC33. Samples from successive fractions (indicated by lane numbers 1–5). **(D)** Purification of His_6 Rep A97C. Samples from column flow-through (lane 1), and successive fractions (indicated by lane numbers 2–8).

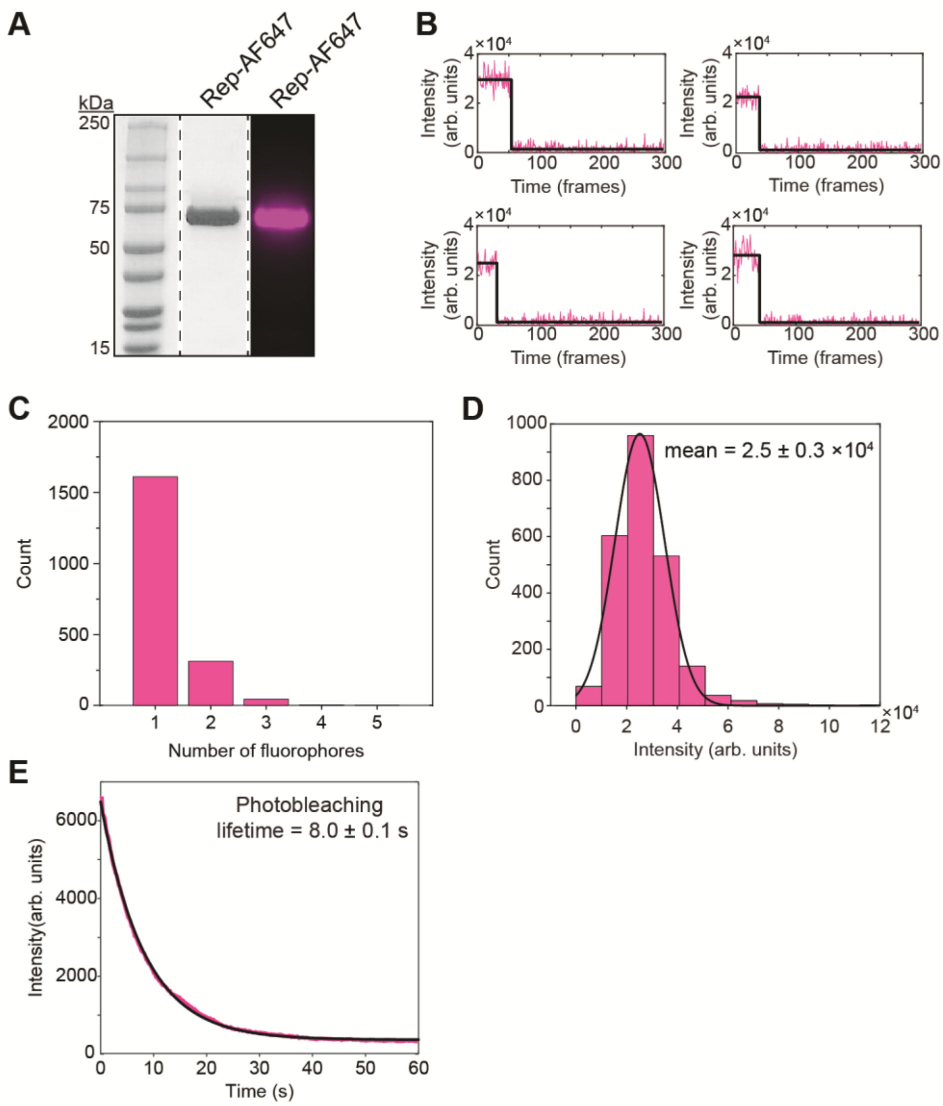

Supplementary Figure S2: Quantification of fluorescent labeling of Rep-AF647. **(A)** SDS-PAGE gel of purified Rep-AF647. Left and middle lanes are stained with Coomassie blue and imaged using a Bio-Rad Gel Doc XR. Right lane is unstained and Alexa Fluor 647 fluorescence imaged using an Amersham Imager 600. **(B)** Example trajectories of Rep proteins deposited on coverslip and subjected to photo-bleaching. Number of fluorophores per Rep monomer are detected by quantifying the single-molecule photobleaching steps using change point analysis (black line). **(C)** Distribution of the number of steps and therefore the number of fluorophores per monomer (*n* = 1483). **(D)** Distribution of the step size of (B) was 2.5 ± 0.3 x 10^4 (mean ± S.E.M., *n* = 1483). **(E)** Average photo-bleaching trajectory for Rep-AF647 (*n* = 1483) at excitation power density of 200 mW cm^{-2}. From a fit with a single-exponential decay function (black line), a photo-bleaching lifetime of 8.0 ± 0.1 s was obtained.

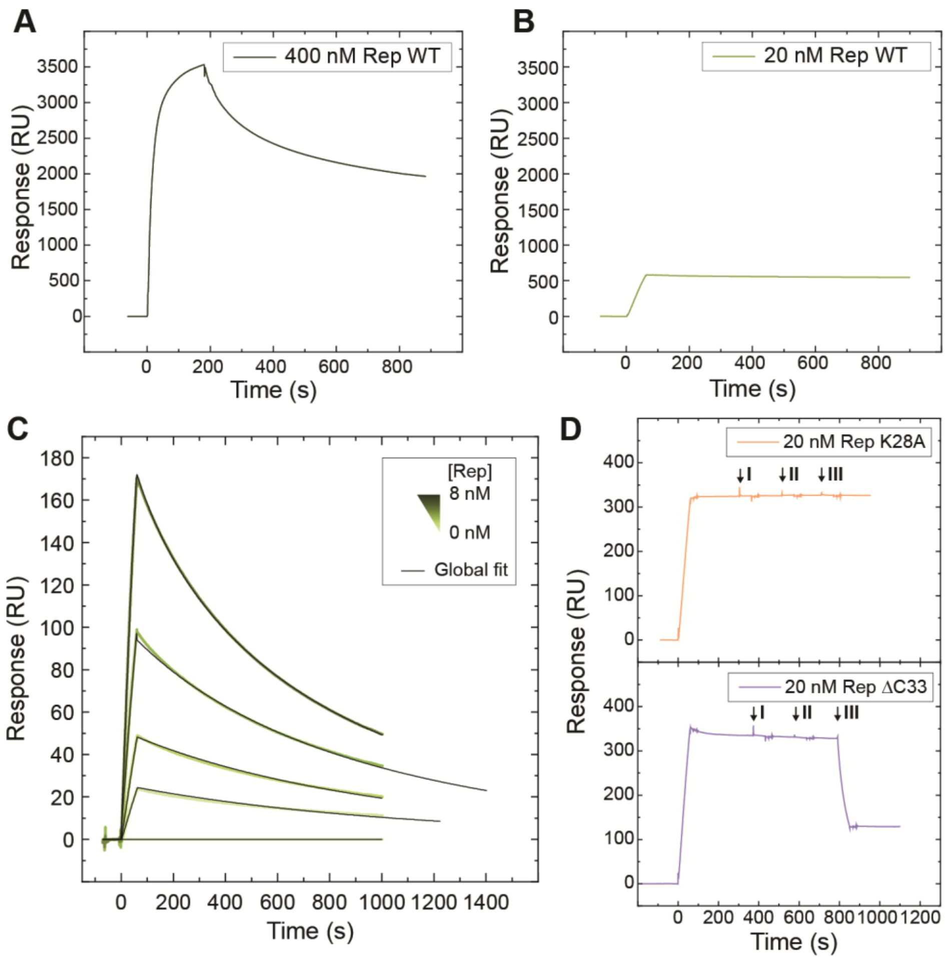

Supplementary Figure S3: Observations of Rep binding to DNA. SPR sensorgrams of 400 nM **(A)** and 20 nM **(B)** Rep WT binding to dT_{35}. **(C)** Association (60 s) and dissociation of titrated (1–8 nM) Rep WT binding to dT_{15} (global fitting of Rep binding (1:1 binding with mass transfer) shown in black yielded a K_D of approximately 500 pM). **(D)** 20 nM Rep K28A (orange – top) and 20 nM Rep ΔC33 (purple – bottom) dissociation after injection of nucleotides AMP-PNP (I), ADP (II) and ATP (III).

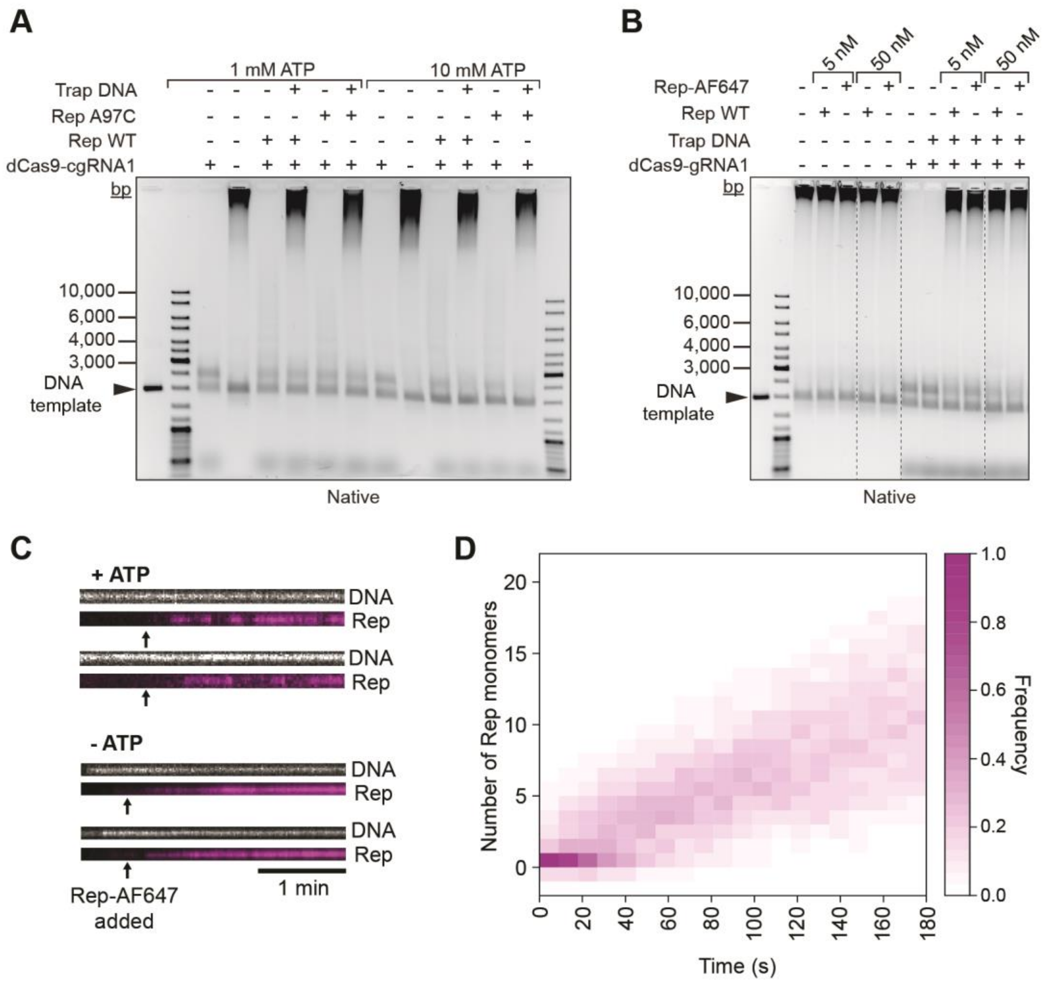

Supplementary Figure S4: Ensemble replication rescue activity tests of Rep A97C and Rep-AF647. **(A)** Rep WT and Rep A97C (50 nM) efficiently remove dCas9-cgRNA1 (50 nM) complex from DNA templates and rescue stalled DNA replication. Note that the appearance of long DNA products signifies efficient replication rescue. A 10-fold increase in ATP concentration (and 2-fold increase in $MgCl_2$ concentration) results in a greater extent of DNA replication rescue, evident by decreased intensity of DNA band at approximately 2.5 kbp. **(B)** Rep-AF647 successfully removes the dCas9-cgRNA1 complex from DNA templates. Irrelevant lanes to the figure are cropped out (dashed lines). **(C)** Example kymographs of Rep-AF647 binding to 2-kbp rolling-circle DNA template bound by SSB and DnaBC, in the presence (top) and absence (bottom) of ATP. Arrows indicate the time point of addition of Rep-AF647 to the flow cell. **(D)** Heatmap of number of Rep-AF647 monomers bound to the DNA template over time in the absence of ATP (*n* = 70).

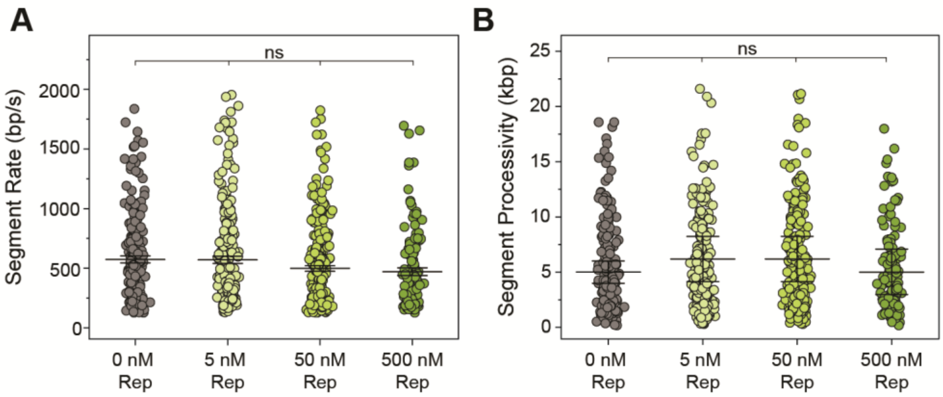

Supplementary Figure S5: Quantification of the effect of Rep WT on the rate and processivity of replication. **(A)** Median rates of replication in the absence (gray) (580 ± 30 bp s^{-1} (median ± S.E.M., n = 179, replication efficiency = 5 ± 1 % (S.E.M.)) and presence of titrated Rep WT (5 nM (light green) (573 ± 30 bp s^{-1} (n = 208, 5 ± 1%), 50 nM (olive green) (500 ± 20 bp s^{-1} (n = 234, 6 ± 1%), and 500 nM (dark green) (470 ± 30 bp s^{-1} (n = 113, 3 ± 1%)), quantified by change-point analysis of single-molecule rolling-circle DNA replication trajectories. **(B)** Mean processivity of replication in the absence (gray) (5 ± 1 kbp (mean ± S.E.M.)) and presence of titrated Rep WT (5 nM (light green) (6 ± 2 kbp), 50 nM (olive green) (6 ± 2 kbp), and 500 nM (dark green) (5 ± 2 kbp)). Mean processivities were determined from fitting a single-exponential decay function to data. Comparison of distributions was conducted using Kruskal-Wallis test for multiple comparisons with Dunn’s procedure where ns indicates no significant difference ($p > 0.05$).

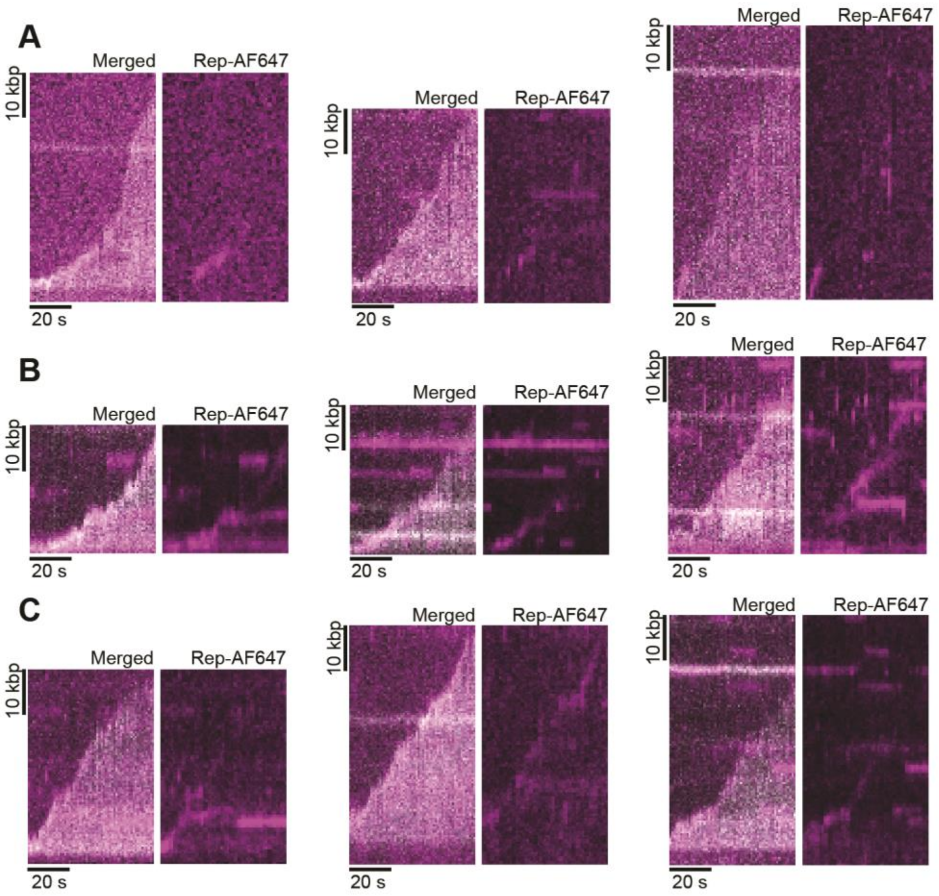

Supplementary Figure S6: Example kymographs of Rep-AF647 during processive rolling-circle DNA replication. Merged kymographs of Rep-AF647 intensity (magenta) and Sytox orange-stained DNA (gray) (left) and Rep-AF647 intensity alone (right). **(A)** 5 nM Rep-AF647 (replication efficiency of 7 ± 1% (S.E.M.)). **(B)** 10 nM Rep-AF647 (5 ± 1%). **(C)** 20 nM Rep-AF647 (4 ± 1%).

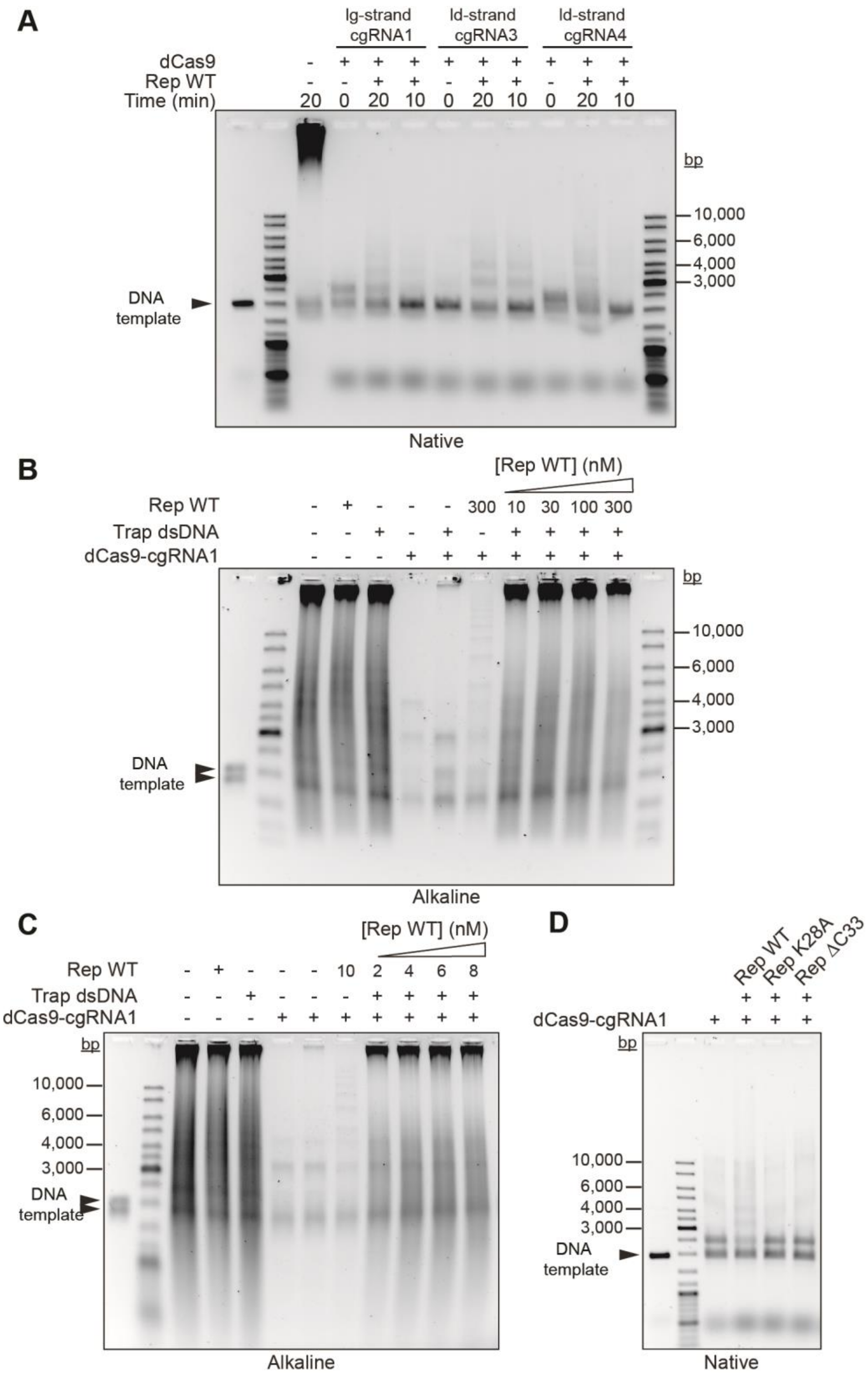

Supplementary Figure S7: Rep rescues DNA replication stalled by dCas9-cgRNA complexes. Reactions contained 200 nM of specified cgRNA and 50 nM dCas9 complex. **(A)** Rep WT rescues stalled DNA replication independent of the DNA strand targeted by the dCas9-cgRNA complex. Rep WT was added to reactions at the 10 min time point. *N* > 2 independent experiments. **(B)** Titration of Rep WT (10–300 nM). Addition of trap dsDNA results in higher extent of rescued DNA products. *N* > 2 independent experiments. **(C)** Titration of Rep WT (2–8 nM). *N* > 2 independent experiments. **(D)** Rep

mutants lacking either the C-terminal domain (ΔC33) or functional ATPase (K28A) cannot rescue dCas9-gRNA1 stalled DNA replication. *N* > 2 independent experiments.

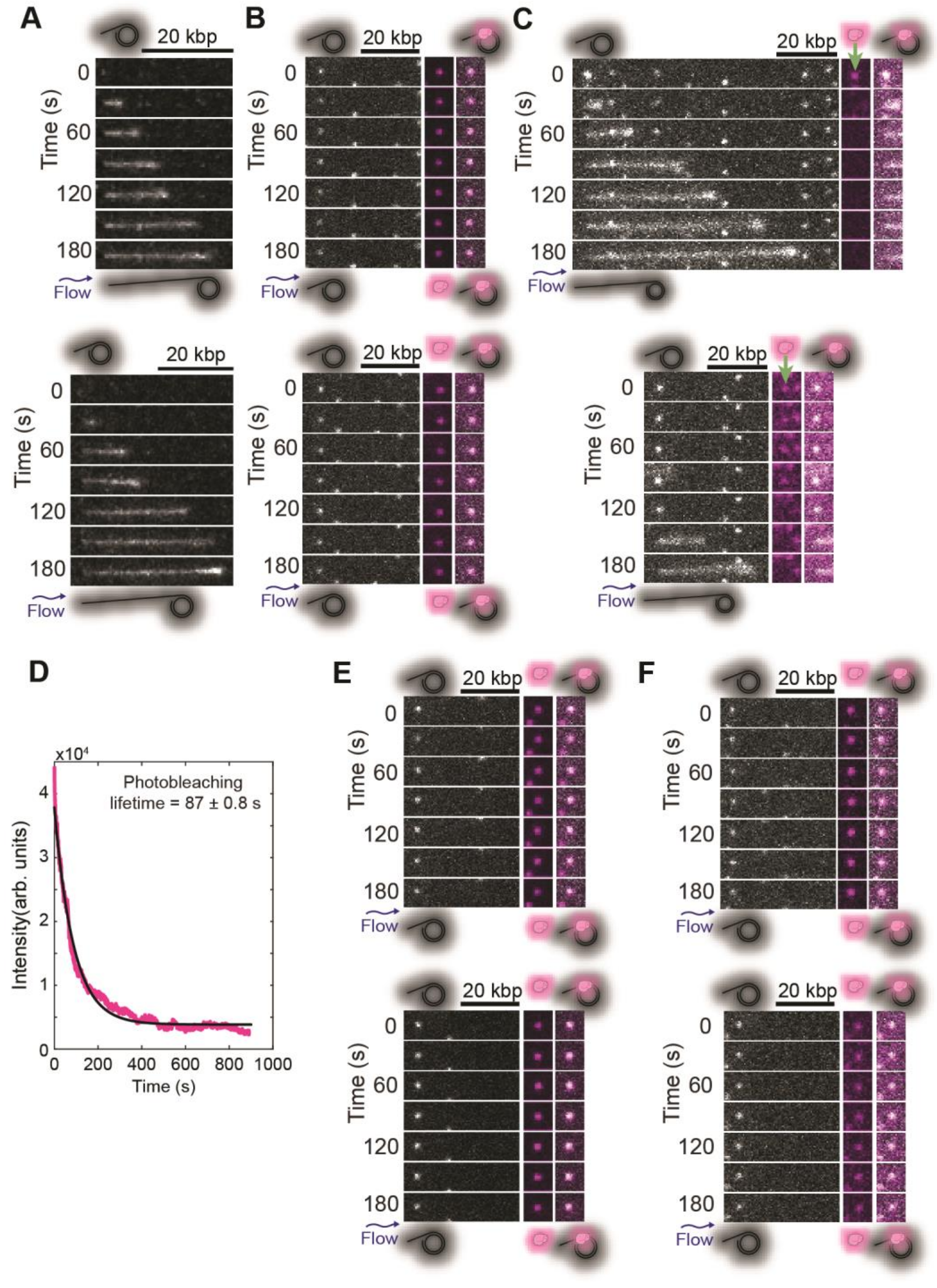

Supplementary Figure S8: Additional example montages of replication rescue of DNA templates (Sytox orange stained; gray scale) pre-incubated with dCas9-cgRNA1-Atto647 (magenta) complexes. **(A)** In the absence of dCas9-cgRNA1 complexes. **(B)** Pre-incubation of the DNA template with dCas9-cgRNA1-Atto647. **(C)** Addition of Rep WT (20 nM) results in disappearance of dCas9-cgRNA1-Atto647 (green arrow). **(D)** Average photo-bleaching trajectory for dCas9-cgRNA1-Atto647 (*n* = 139) at excitation power density of 200 mW cm^{-2}, imaged every 200 ms. Single-exponential decay fit (black

line) revealed a photo-bleaching lifetime of 87 ± 1 s. **(E)** Rep K28A and **(F)** Rep ΔC33 cannot remove dCas9-cgRNA1-Atto647 complexes.

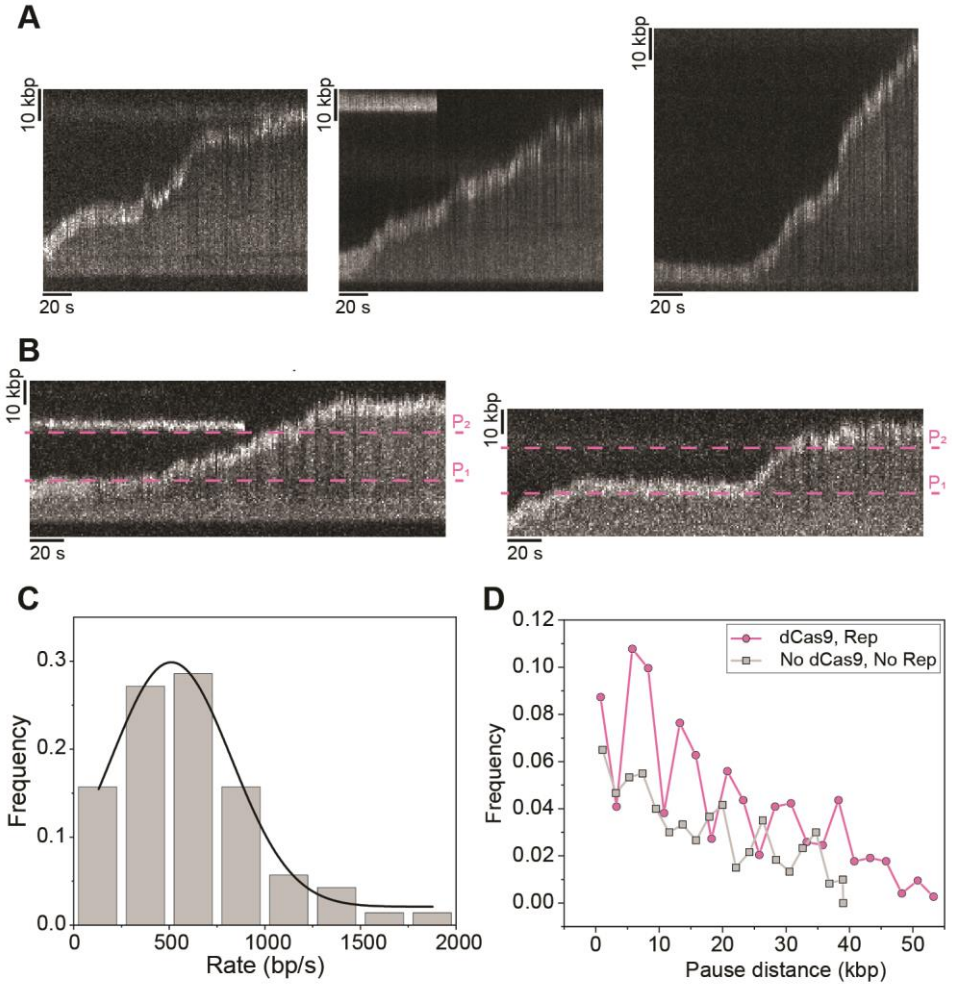

Supplementary Figure S9: Single-molecule rolling-circle DNA replication of an 18-kbp DNA template. **(A)** Three example kymographs of elongating DNA replication of Sytox orange-stained 18-kbp rolling-circle DNA template (gray) (*n* = 32 molecules; replication efficiency of 7 ± 1% (S.E.M.)). Large circle of template is resolved at the tip of the replicating molecule at a higher intensity as it is stretched out by flow. **(B)** Two example kymographs of elongating DNA replication showing multiple pausing and rescue events by dCas9-cgRNA1 (0.25 nM) and Rep WT (10 nM) in solution (*n* = 26 molecules; 3 ± 1%). The dashed lines (magenta) indicate the theoretical pause start sites at approximately 17 kbp (P_1) and 36 kbp (P_2). **(C)** Histograms of the rate of replication for 18-kbp rolling-circle DNA templates (520 ± 130 bp s^{-1}, *n* = 70) as in (A), fit to a Gaussian distribution. **(D)** Pairwise distance analysis of the paused start sites of 18 kbp replication rescue events in the presence of 10 nM Rep-AF647 and 0.25 nM dCas9-cgRNA1 (*n* = 37 pauses/733 kbp) (magenta) and absence (*n* = 28 pauses/600 kbp) (gray) for the first 60 kbp of DNA products.

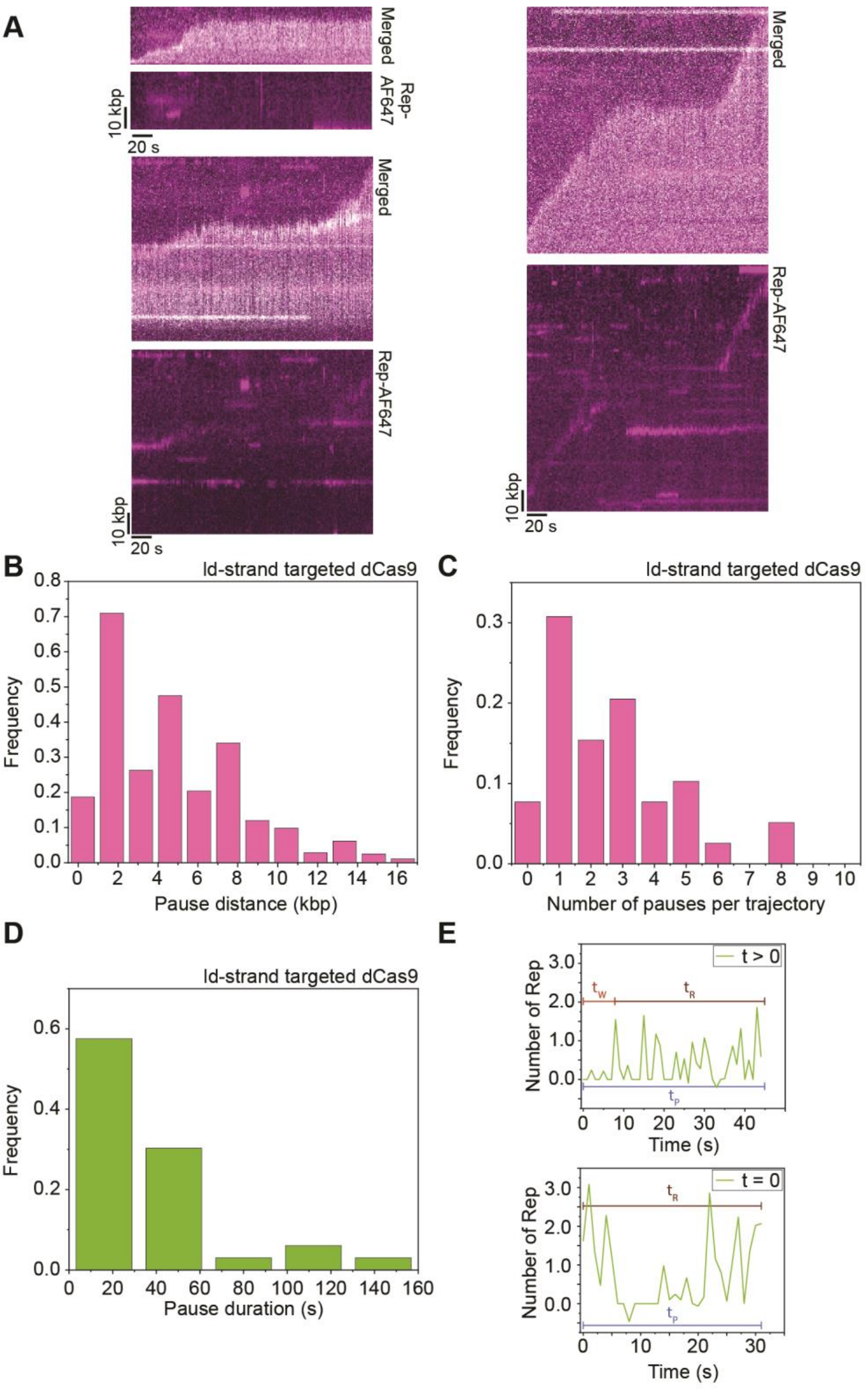

Supplementary Figure S10: Single-molecule replication rescue of 2-kbp rolling-circle DNA templates. **(A)** Three example kymographs of rolling-circle DNA replication pausing and rescue events in reactions containing dCas9-cgRNA1 (0.25 nM) and Rep-AF647 (10 nM). Merged kymographs of Sytox orange-stained DNA products (gray) and Rep-AF647 (magenta) (top), and Rep-AF647 intensity alone (bottom). (Replication efficiency of 3 ± 1%). (B–D) Assays containing leading-strand target dCas9-cgRNA4 complexes (0.25 nM) and Rep-AF647 (10 nM) show **(B)** periodicity of pausing and rescue events (*n* = 43 pauses/358 kbp), **(C)** number of pausing events per replicating molecule (*n* = 40 molecules;

replication efficiency of 2 ± 1%), and **(D)** mean duration of pauses of 40 ± 20 s (n = 33 pauses). **(E)** Annotated example of Rep-AF647 intensity traces over time showing the time points used for determining the pause duration (t_P – blue), association wait time (t_R – red) and pause resolve time (t_R – dark brown) for each Rep activity (t > 0, left; t = 0, right).

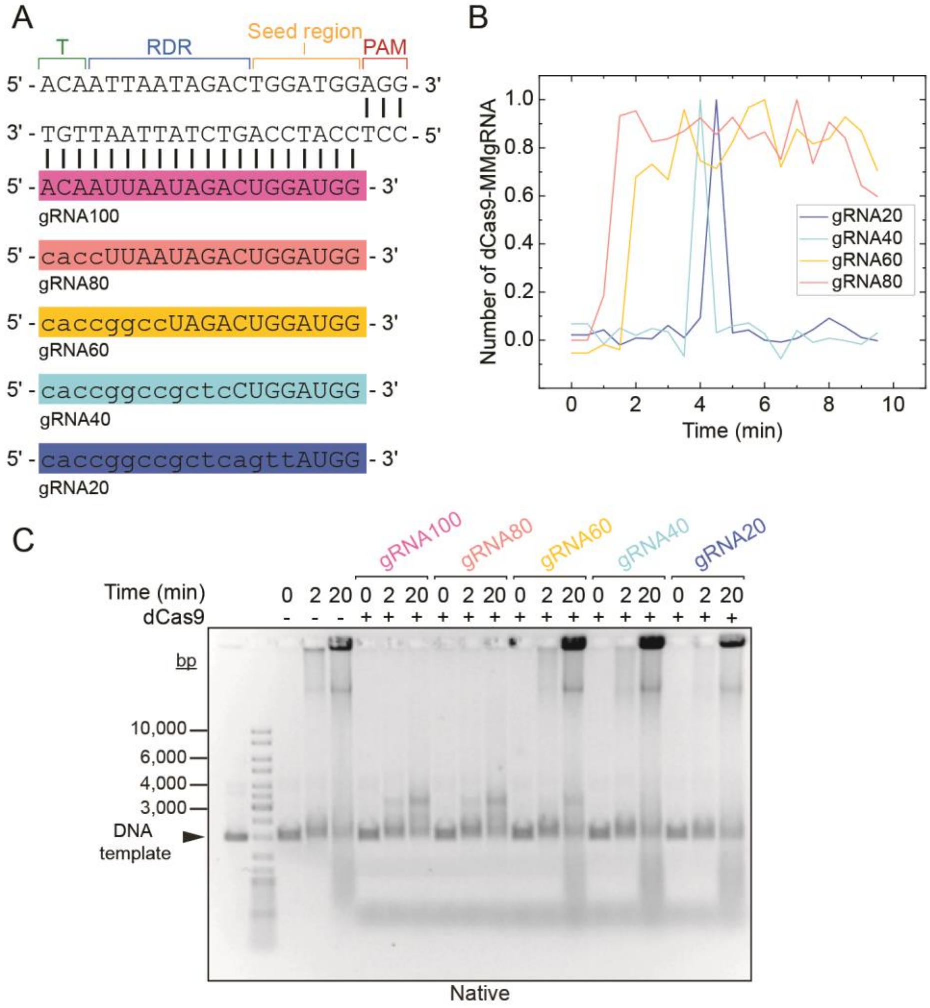

Supplementary Figure S11: Characterization of dCas9 complexed with mismatch gRNAs. **(A)** Designs of cgRNA1 and various mismatch gRNAs ranging from 80–20% complementarity to the target sequence of cgRNA1 (or gRNA100). Mismatches, denoted by lowercase letters, span across the DNA-RNA hybrid from the PAM distal region (or terminal region (T – green)), reversibility-determining region (RDR – blue), seed region (yellow) and to the PAM proximal region (red). **(B)** Example lifetime intensity trajectories of dCas9-gRNA80 (peach), dCas9-gRNA60 (yellow), dCas9-gRNA40 (light blue) and dCas9-gRNA20 (blue). Intensity is corrected for the intensity of a single dCas9-gRNA-Atto647 molecule measured by photobleaching analysis. **(C)** Time course ensemble characterization of dCas9-MMgRNA complexes blocking rolling-circle DNA replication over 20 min in the absence of Rep proteins and trap dsDNA.
